# Optimization of viral resuspension methods for carbon-rich soils along a permafrost thaw gradient

**DOI:** 10.7717/peerj.1999

**Published:** 2016-05-17

**Authors:** Gareth Trubl, Natalie Solonenko, Lauren Chittick, Sergei A. Solonenko, Virginia I. Rich, Matthew B. Sullivan

**Affiliations:** 1 Department of Soil, Water and Environmental Science, University of Arizona, Tucson, AZ, United States; 2 Current affiliation: Department of Microbiology, Ohio State University, Columbus, OH, United States; 3 Department of Ecology and Evolutionary Biology, University of Arizona, Tucson, AZ, United States; 4 Current affiliation: Department of Ecology, Evolution and Organismal Biology, Ohio State University, Columbus, OH, United States; 5 Current affiliation: Department of Civil, Environmental and Geodetic Engineering, Ohio State University, Columbus, OH, United States

**Keywords:** Viral diversity, Viral ecology, Soil viruses, Phages, Microbial ecology, Humic-laden, Permafrost, Viral methods, Active layer, Peatland

## Abstract

Permafrost stores approximately 50% of global soil carbon (C) in a frozen form; it is thawing rapidly under climate change, and little is known about viral communities in these soils or their roles in C cycling. In permafrost soils, microorganisms contribute significantly to C cycling, and characterizing them has recently been shown to improve prediction of ecosystem function. In other ecosystems, viruses have broad ecosystem and community impacts ranging from host cell mortality and organic matter cycling to horizontal gene transfer and reprogramming of core microbial metabolisms. Here we developed an optimized protocol to extract viruses from three types of high organic-matter peatland soils across a permafrost thaw gradient (palsa, moss-dominated bog, and sedge-dominated fen). Three separate experiments were used to evaluate the impact of chemical buffers, physical dispersion, storage conditions, and concentration and purification methods on viral yields. The most successful protocol, amended potassium citrate buffer with bead-beating or vortexing and BSA, yielded on average as much as 2-fold more virus-like particles (VLPs) g^−1^ of soil than other methods tested. All method combinations yielded VLPs g^−1^ of soil on the 10^8^ order of magnitude across all three soil types. The different storage and concentration methods did not yield significantly more VLPs g^−1^ of soil among the soil types. This research provides much-needed guidelines for resuspending viruses from soils, specifically carbon-rich soils, paving the way for incorporating viruses into soil ecology studies.

## Introduction

Global carbon (C) cycling is intensively studied and modeled partly due to its impact on climate change (IPCC, 2014). About 50% of global soil C (∼1,300 Pg; Hugelius et al., 2014; Schuur et al., 2015) is stored frozen in permafrost soils, which underlain ∼25% of Earth’s land surface (Graham et al., 2012). Problematically, permafrost soils are thawing at a rate of >1 cm yr^−1^ (Elberling et al., 2013) and predicted to have ∼85% loss (according to RCP 8.5 from IPCC, 2014) by the end of the 21st century (Slater & Lawrence, 2013). Further, the rate of permafrost thaw is highly dependent on the location’s environmental parameters, with some habitats experiencing abrupt thaw and others a slower thaw progression (Jansson & Taş, 2014; Abbott & Jones, 2015; Schuur et al., 2015). The fate of the thawing organic matter is a key unknown for improving predictive models of climate change (Tarnocai et al., 2009; Schuur et al., 2015).

Permafrost and active layer (the seasonally-thawed soils overlying permafrost) C cycling is mediated by diverse microorganisms including many novel phylotypes (Prater, Chanton & Whiting, 2007; Schimel, Balser & Wallenstein, 2007; Mackelprang et al., 2011; Jansson & Taş, 2014; Frank-Fahle et al., 2014). Active layer microbiota are likely most relevant to understanding C cycling during ongoing thaw, since the deeper habitat’s characteristics are becoming more like those of the active layer under climate change (i.e., seasonally thawed, more hydrologically connected, and more plant-impacted). Microorganisms in the active layer of permafrost soils reach biomasses comparable to those in temperate soils (Hansen et al., 2007; Frank-Fahle et al., 2014) with microbial diversity highest in the surface and decreasing with depth (Frank-Fahle et al., 2014). Resident microbiota have numerous roles in soil carbon processing including degradation of complex molecules, fermentation, and methane cycling (e.g., Hultman et al., 2015), and have been quantitatively linked to aspects of ecosystems’ carbon gas emissions (McCalley et al., 2014).

While the key roles that microorganisms play in biogeochemical cycling in soils are now widely recognized (e.g., Paul, 2014), virtually nothing is known about the viruses that infect these microorganisms. In the oceans, where viruses have been intensively studied, they are known to be major modulators of microbial metabolic outputs and ecosystem function. Marine viruses are abundant and dynamic, with viral-caused host mortality lysing ∼1/3 of host cells per day, and horizontal gene transfer moving ∼10^29^ genes per day (reviewed in Fuhrman, 1999; Paul, 1999; Wommack & Colwell, 2000; Weinbauer, 2004; Suttle, 2007). These viral caused events liberate C and nutrients, and impact global ocean C cycling (Suttle, 2005). Marine viruses also alter C cycling by manipulating core microbial metabolisms of their hosts via metabolic reprogramming during infection, which now includes viral-encoded genes for photosynthesis, central C metabolism, and sulfur cycling (Sullivan et al., 2006; Roux et al., 2014; Anantharaman et al., 2014; Hurwitz, Brum & Sullivan, 2015). Recent discoveries have been aided by the development of a quantitative sample-to-sequence pipeline for studying double-stranded DNA viral communities via metagenomics (Duhaime et al., 2012; Solonenko & Sullivan, 2013). Such quantitative viral metagenomes (viromes) have enabled the formation of systematic ocean virome datasets (Hurwitz & Sullivan, 2013; Brum et al., 2015), revealing extensive new biology ranging from estimating the size of the global virosphere to documenting “core” and “flexible” genes of varied ocean viral communities (reviewed in Brum & Sullivan, 2015).

While viruses likely play similarly important roles in soils, advances in soil viral ecology have been relatively limited, due to the technical challenges of working with soils’ variable physicochemistry (i.e., pedodiversity). Even the first step in studying viruses, accessing and separating viral particles from the complex soil matrices, is daunting because >90% of viruses tend to adsorb to soil particles (Goyal & Gerba, 1979; Moore et al., 1981; Moore et al., 1982). Resuspension of viruses from soils has thus been attempted by varying chemical resuspension and physical dispersion methods. On the chemical side, many resuspension buffers have been utilized, including 10% beef extract, 250 mM glycine buffer, 10 mM sodium pyrophosphate (all used in e.g., Williamson, Wommack & Radosevich, 2003), 1% potassium citrate (Paul et al., 1993; Williamson, Wommack & Radosevich, 2003), and sodium deoxycholate (Williamson et al., 2013). To aid in resuspension, physical dispersion methods have been implemented, including vortexing (Ashelford, Day & Fry, 2003), sonication (Duhamel & Jacquet, 2006; Danovaro & Middelboe, 2010), bead-beating (Ashelford, Day & Fry, 2003; Williamson et al., 2013), mechanical homogenization (Willner et al., 2009), and blending (Williamson et al., 2007; Swanson et al., 2009). These methods have been explored across diverse soil and sediment environments including freshwater sediment (Danovaro & Middelboe, 2010) and different types of soil (Williamson, Radosevich & Wommack, 2005; Williamson et al., 2013). Viruses from a peatland have been obtained once, but without methodological optimization (Quaiser et al., 2015). No universal solution has been identified, and the comparisons of methods across different soils have indicated that one is unlikely to be found (unsurprisingly given the physicochemical diversity of soils). Therefore, this first, crucial step of extracting viruses from soils remains a major bottleneck for studying soil viral ecology.

In spite of these challenges, there is some evidence that viruses impact soil microorganisms and their ecosystem outputs. Metagenomic studies in rainforest soils indicate that viral species richness is greater than bacterial species richness by an order of magnitude where moisture content and OM are high (Fierer et al., 2007; reviewed in Kimura et al., 2008). Additionally, viral diversity may be greater in soils than in marine water column environments, because soil microorganisms are thought to be more diverse than marine microorganisms (Torsvik, Øvreås & Thingstad, 2002; Weinbauer & Rassoulzadegan, 2004; Smalla & Van Elsas, 2010; Tamames et al., 2010; Crump, Amaral-Zettler & Kling, 2012), and viral and microbial diversity are correlated (Maranger & Bird, 1995; Anesio et al., 2004; Ballaud et al., 2015). In addition, soil viruses have clear seasonal population dynamics with different temporal and spatial distributions (Ashelford et al., 1999; Ashelford et al., 2000; Ballaud et al., 2015), which could act as mechanisms for maintaining the coexistence of a diverse community (Chesson, 2000). Still, little is known about how soil viruses contribute to natural ecosystem function because prior work was motivated by agronomical and epidemiological research (Kimura et al., 2008).

High organic matter soils rich in humic acids are an ecologically critical soil type (representing diverse wetlands, for example, including peatlands) and likely interact with viruses quite differently from mineral soils, due to the different particle chemistry (i.e., the diverse organic acids can form complexes with charged viral capsids; Stevenson, 1994). In addition, humics can co-extract viruses, making quantification difficult. Here we present three separate experiments to evaluate the effects of buffers, physical dispersion, storage conditions, and concentration and purification methods on virus yields from three peat soil types. Specifically, we optimized extraction conditions for shallow (1–5 cm) and mid-depth (36–40 cm) permafrost-associated peat soils (ranging from 36–100% organic matter), a particularly climatically important soil type due to ongoing thaw from climate change, as described above. The active layer soils span three stages of permafrost thaw in Stordalen Mire, northern Sweden, and vary physicochemically (Hodgkins et al., 2014) and biologically (Mondav et al., 2014; McCalley et al., 2014). Water content and active layer depth increase along the permafrost thaw gradient, and OM becomes more labile, while pH ranges from 4.0 (bog) to 6.0 (fen) (Hodgkins et al., 2014). The dominant microbiota include Acidobacteria, Proteobacteria and Euryarchaeota, the latter including both hydrogenotrophic and acetoclastic methanogens (Mondav et al., 2014; McCalley et al., 2014).

## Methods

### Sample collection

Peatland soil cores were collected in July 2013 and July 2014 from palsa (raised hummock underlain by intact permafrost), moss-dominated (*Sphagnum* spp.) bog, and sedge-dominated (Eriophorum) fen habitats in Stordalen Mire, in Abisko, northern Sweden. Further description of the study site and habitat classification is reported in Hodgkins et al. (2014). Soil was gathered with an 11 cm-diameter custom circular push corer at palsa sites, and with a 10 cm × 10 cm square Wardenaar corer (Eijkelkamp, The Netherlands) at the bog and fen sites. Each core was carefully removed from the coring device, while maintaining the integrity of the core and its layers, and placed on a sterile surface. A measuring tape and camera were used to measure and document the core. Using a sterile knife, the outer 1 cm of the core was removed and then the knife was re-sterilized (cleaned with 70% ethanol after each use) and used to cut the core in five centimeter increments from 1 to 40 cms (if applicable). Intervals were placed in 50 ml conical tubes and treated in one of two ways, per “storage” methods below. Samples were transported to nearby Abisko Naturvetenskapliga Station, and then to the University of Arizona for processing.

### Experimental overview

Three sets of experiments evaluated the impact of buffers, physical dispersion, storage conditions, and concentration and purification methods on viral yields. Experiment 1A/B used 2013 samples, pooled from 1–40 cm (evenly) of each soil core that was refrigerated at 4 °C during transportation (∼1–2 week storage while at the field site and then ∼1 week for transportation to Arizona) and then either stored at −80 °C or 4 °C. The 2013 samples were used to test chemical and physical dispersion methods. Experiment 2 used 2014 samples from two depths and tested frozen versus chilled storage methods. Experiment 3 was an extension of Experiment 2, with the same samples, and tested the efficacy of a common concentration and purification method.

The effectiveness of each method was quantified by epifluorescence microscopy (EFM), to determine the viral abundance of each sample. EFM is considered to be a better enumeration technique than plaque assays, flow cytometry, and transmission electron microscopy (TEM), because it is the most reliable, cost effective, and readily available (Hermes & Suttle, 1995; Weinbauer & Suttle, 1997; Noble & Fuhrman, 1998; Ashelford, Day & Fry, 2003; Brum, Schenck & Sullivan, 2013; Williamson et al., 2013). Samples were mixed with SYBR Gold, ascorbic acid anti-fade solution, and beads of a known concentration (as in Cunningham et al., 2015). Counts of virus-like particles and beads were compared to enumerate the viruses (Cunningham et al., 2015). All treatment comparisons of viral yields were tested for significance using two-tailed paired *t*-tests. Additional viral characterization was obtained by analysis of VLPs via TEM from 10 fields of view for each sample using previously describe preparation techniques (Fig. S1; Brum, Schenck & Sullivan, 2013).

#### Experiment 1A: optimizing resuspension buffers

The purpose of this experiment was to determine the best buffer for resuspending viruses from peatland soils. Three chemical desorption buffers were used: 1% potassium citrate (“KC”; Paul et al., 1993; Williamson, Wommack & Radosevich, 2003), amended 1% potassium citrate (“AKC”; derived from Hewson & Fuhrman, 2003), and amended 5 mM sodium pyrophosphate (“PP”; Middelboe, Glud & Finster, 2003). AKC was 1% potassium citrate amended with 10% phosphate buffered-saline (PBS), 5 mM ethylenediaminetetraacetic acid (EDTA), and 150 mM magnesium sulfate (MgSO_4_). The PP was 5 mM sodium pyrophosphate, 10% Phosphate buffered saline, 5 mM EDTA, and 150 mM MgSO_4_.

Each sample was thawed at room temperature (∼23 °C), and 5 ± 1 g were weighed out into 50 mL super-speed centrifuge tubes (Thermo Fisher Scientific Inc. Waltham, MA, USA), and 10 mL of a buffer was added. The tubes were placed horizontally on a platform shaker and shaken at 400 rpm for 15 min at 4 °C.

Sonication was then used as a physical dispersion method for all of Experiment 1A samples, following the protocol of Williamson, Wommack & Radosevich (2003). Tubes were inserted into a styrofoam 50 mL tube rack with the bottoms pushed out, such that the styrofoam holder acted as a floatation device, and added to a sonicator (Branson 2510, Branson Ultrasonics Corp, Danbury, CT, USA) containing deionized water at 4 °C in a walk-in refrigerator. The samples were sonicated at 42 kHz for one minute, followed by 30 s of manual shaking; these two steps were then repeated twice more (per Williamson, Wommack & Radosevich, 2003).

The samples were then centrifuged for 20 min at 15,000 g at 4 °C to pellet debris, and the supernatant was transferred into a new 50 ml tube. Then, following previous evidence that sequential re-extraction of the initial soil maximizes virus recovery (Williamson, Radosevich & Wommack, 2005), the pelleted debris was re-resuspended, beginning again at the initial addition of 10 ml of buffer and continuing through shaking and sonication. The pellet was then re-resuspended a third and final time. The final filtrates for each sample were combined into one 50 mL tube. If there was visible debris in the supernatant, we then filtered it through a 0.45 µm vacuum filter (Corning, Corning, NY, USA) into 50 mL tubes. The final filtrates for each sample were combined into one 50 mL tube. Samples were stored at 4 °C until enumerated (within a week).

#### Experiment 1B: physical dispersion methods

For this experiment, we tested physical dispersion methods on viruses from peatland soils. We used sonication in experiment 1A, and wanted to test those results against vortexing (Williamson, Radosevich & Wommack, 2005; Helton, Liu & Wommack, 2006) and bead-beating (Ashelford, Day & Fry, 2003; Williamson et al., 2013). We used 2013 samples mixed and stored the same as experiment 1A, and processed using AKC buffer. We followed the same protocol as before, except rather than sonicating, both vortexing and bead-beating were tested. Vortexing was on high for one minute, followed by 30 s of manual shaking using vortex adapters. Bead-beating involved the addition of 1.65 g of 1.4 mm ceramic beads (33% bead weight to sample weight; bead size was chosen to shear plant material) to each tube, then vortexing on high for one minute, followed by 30 s of manual shaking using vortex adapters. The samples were centrifuged, supernatants were collected, and pellets were re-resuspended two additional times as in Experiment 1A. Supernatants were pooled among sequential resuspensions and filtered, and particles were counted by EFM as previously described.

#### Experiment 2: optimization of storage conditions

The first group of experiments was performed in duplicate to assess which buffer and physical dispersion method worked best before the 2014 field season. With the new methods in mind, we collected more soil samples from all three habitats and tested two distinct storage methods, while also comparing protocol success on peats from two depths (1–5 cm, known as “shallow”, and 36–40 cm, known as “deep”). In 2014, two sets of samples were collected in triplicate and one set was immediately flash frozen in liquid nitrogen and stored at −80 °C, while the other was chilled at 4 °C upon collection and throughout storage (∼1–2 week storage while at field site, ∼1 week for transportation to Arizona, and then samples were processed and enumerated within a week).

All samples were weighed and moved into 50 mL super-speed centrifuge tubes as previously described, now in triplicate. Based on results from the initial experiments, we utilized AKC buffer and vortexing for virus resuspension. The samples were centrifuged, filtered, and a total of three resuspensions were performed before particles were counted under EFM as previously described.

#### Experiment 3: optimization of concentration and purification methods

We used deep samples from experiment 2 testing the effect bovine serum albumin (BSA) had on viral adsorption to 100 kDa Amicon filters (EMD Millipore, Darmstadt, Germany) and then CsCl purification. Amicon filter membranes were tested with and without coating with 2 mL of 1% BSA (w/v) in PBS to reduce adhesion of viruses to the membrane (Deng et al., 2012). The resulting viral concentrates were layered each onto a CsCl step gradient (1.65, 1.5, 1.4, 1.2 g ml^−1^ in sterile water, *sensu* Thurber et al., 2009), then centrifuged in an SW41 rotor (Beckman) at 24,000 rpm (102,000 g) for 2 h at 4 °C. Fractions were collected by piercing the bottom of the desired density layer with a needle. Droplets were collected into in 0.5 ml volumes for a total of 7 fractions. Viruses were harvested from fractions with densities of 1.4–1.52 g ml^−1^.

## Results and Discussion

**Figure 1:**
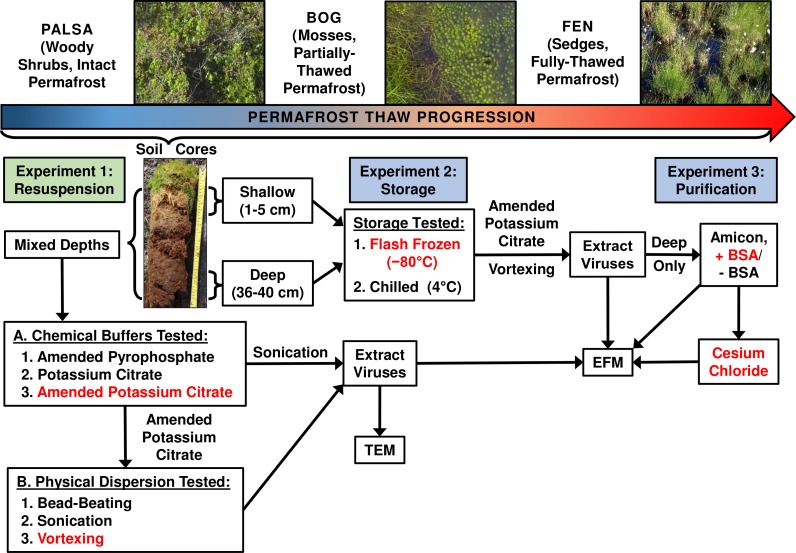
Optimizing viral recovery. Three experiments tested resuspension, storage, and purification conditions for viral recovery on a range of peatland soils from this thawing permafrost site. Red font color indicates the best-performing option within each set. EFM, epifluorescence microscopy; TEM, transmission electron microscopy; BSA, bovine serum albumin. Experiment 1 used peatland samples from 2013 and Experiments’ 2 & 3 used peatland samples from 2014. Palsa and bog pictures contributed by Anthony Garnello.

Three separate experiments were used to evaluate the impact of chemical buffers, physical dispersion, storage conditions, and concentration and purification methods on viral yields from three distinct permafrost soil types along a thaw gradient (overview in Fig. 1).

### Experiment 1A: optimizing resuspension buffers

**Figure 2:**
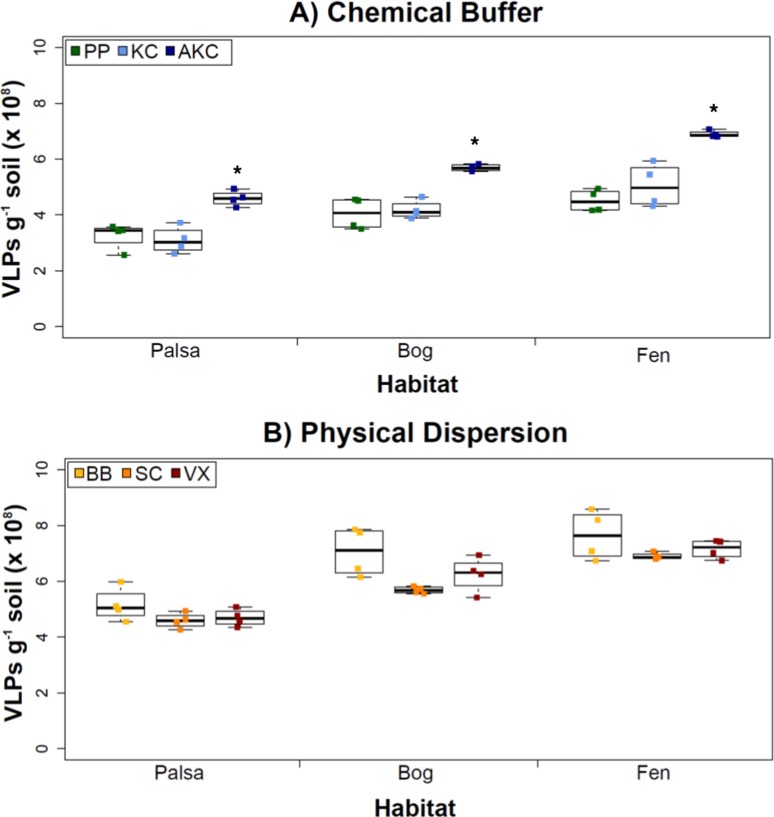
The impact of buffers and physical dispersion methods on viral yields. (A) Viral yields from different buffers (Experiment 1A). PP, sodium pyrophosphate; KC, potassium citrate; AKC, amended potassium citrate. Each treatment was followed by sonication for physical dispersion. (B) Viral yields from different physical dispersion methods (Experiment 1B). VX, vortexing; BB, bead-beating; SC, sonication. Each replicate was in AKC buffer. An ^∗^ denotes statistically significant difference (*p* < 0.05) within the soil type.

The first step in obtaining viruses from soils is to efficiently resuspend them in a buffer that reduces chemical and electrostatic bonds (Dowd et al., 1998; Michen & Graule, 2010), neutralizes pH (Dowd et al., 1998), normalizes moisture content (Williamson, Radosevich & Wommack, 2005), and stabilizes viral capsids (Hurst, 1988). It has been suggested that isoelectric point and hydrophobicity of individual virus capsids are the most important factors influencing how they interact with soils (Dowd et al., 1998; Williamson, Wommack & Radosevich, 2003). We tested a diversity of buffers that were most promising from the literature in other soil systems to maximize viral yields. Specifically, three permafrost-associated peatland soils were used to compare VLP yields after resuspension in each of three buffers (KC, AKC, and PP) with the soils interrogated by a single physical dispersion method (Fig. 2A). We developed buffer AKC to control for changing chemistry (e.g., changing pH and OM chemistry and concentrations) across the Stordalen Mire soils (Hodgkins et al., 2014): we amended KC with 10% PBS (which helps maintain pH and osmotic balance; Sperelakis, 2012), 5 mM EDTA (a ligand and chelating agent that helps prevent environmental interference; Conway et al., 1999), and 150 mM MgSO_4_ (used to stabilize virus capsids; Speir et al., 2006).

Overall, the AKC buffer yielded significantly more VLPs g^−1^ soil than buffers KC or PP (*p* < 0.05, two-tailed paired *t*-test; Fig. 2A). For all buffers tested, VLP yields increased from palsa to bog and then to fen, with fen soils having up to 72% more VLPs than palsa soils (Fig. 2A). This is consistent with previous observations of soil VLP abundance correlating with soil moisture content (Williamson, Radosevich & Wommack, 2005; reviewed in Kimura et al., 2008); here palsas are driest, bogs have a fluctuating water table, and fens are fully inundated. Other factors vary progressively along the thaw gradient too (notably soil OM content and lability) and may also be influencing VLP abundances and/or recoveries (Hodgkins et al., 2014). The VLP counts, of ∼5–7 × 10^8^ VLPs g^−1^ soil, were in the same order of magnitude as or higher than previously reported using KC across wetland, clay, dune, forest, and park soils enumerated with the same epifluorescence microscopy technique (Williamson et al., 2013). (In that study, KC gave higher viral recoveries across soil types than water or sodium deoxycholate as a buffer; Williamson et al., 2013).

### Experiment 1B: Physical dispersion methods

With an empirically optimized resuspension buffer, AKC, we next explored how physical dispersion methods (all with AKC) impacted viral yields across our three soil types. These three methods were chosen as follows: the sonication method has been most heavily tested in the literature, the beads in bead-beating might help with desorption of viruses (both reviewed in Kimura et al., 2008), and vortexing might lead to the least tail breakage (Williamson, Helton & Wommack, 2012).

However, in our samples, Experiment 1B revealed no statistically significant difference in VLP yields across the physical dispersion methods tested (Fig. 2B). VLP counts ranged from 4.59 × 10^8^ (±2.75 × 10^7^) VLPs g^−1^ soil for sonication in palsa to 7.65 × 10^8^ (±8.83 ×10^7^) VLPs g^−1^ soil for bead-beating in fen. As in Experiment 1A’s results, VLP yields were again lowest in palsa and highest in fen. Although bead-beating was not significantly better, it did have on average higher VLPs than the other physical dispersion methods. We hypothesize shearing of the humic material helped viruses desorb from the peatland soil.

While no significant differences across the methods were detected, we note that there are many variations of each method reported in the literature, which were not tested here. Variants of the sonication method include storing the samples on ice during sonication (Duhamel & Jacquet, 2006; Danovaro & Middelboe, 2010) and varying the sonication times (Danovaro & Middelboe, 2010). Sonication has been the most widely used method across sediment and soil environments and has performed better when combined with a well-paired resuspension buffer such as the KC buffer examined in Experiment 1A (Williamson, Wommack & Radosevich, 2003; Duhamel & Jacquet, 2006; Danovaro & Middelboe, 2010). Bead-beating methods have also been used frequently (Ashelford, Day & Fry, 2003; Williamson et al., 2013), but there is no evidence for its increased efficacy over other methods. However, not all types and sizes of beads have been tested. In our case, vortexing was performed using the same equipment and method as bead-beating, but without beads. Vortexers come with many different speed settings and attachments, and each could potentially influence viral yields. Finally, while vortexing is thought to reduce (by 20%) tail breakage as compared to sonication methods (Williamson, Helton & Wommack, 2012), we did not investigate this further since our goals were largely to obtain VLPs for metagenomic sequencing.

**Figure 3:**
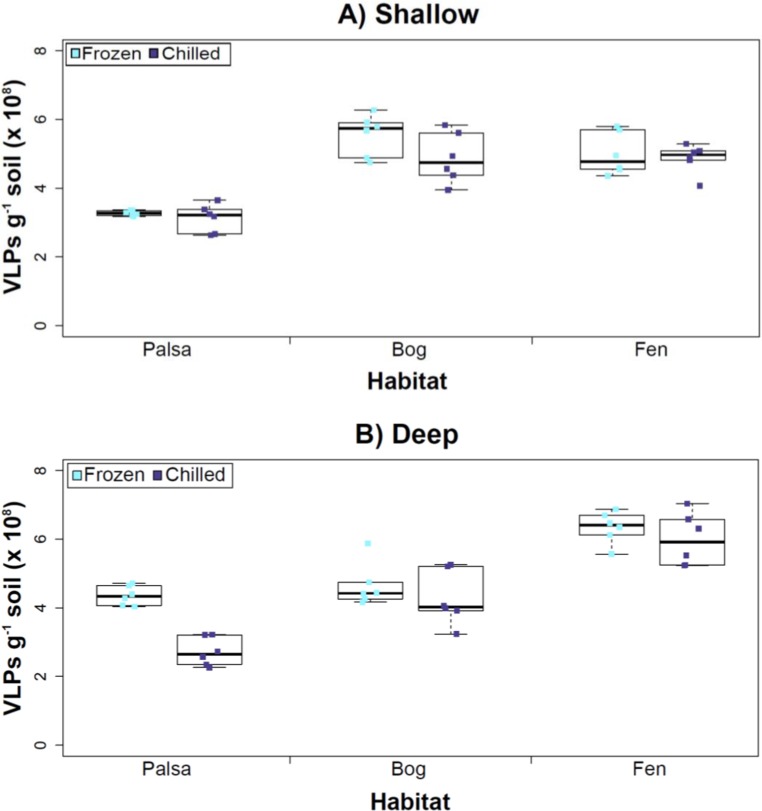
The impact of storage conditions on viral yields for different depth soils. Samples from shallow (1–5 cm; A) or deep (36–40 cm; B) depths, and stored frozen (flash frozen and kept at −80 °C) or chilled (4 °C), were counted using epifluorescence microscopy.

### Experiment 2: optimization of sampling conditions

After Experiments 1A and 1B, we sought to better understand how sampling and storage conditions impacted viral yields across multiple depths. We used AKC buffer and vortexing to resuspend the viruses and examined the effect storage temperature had on VLP abundance. Viral abundances in soils may change with depth like microbial biomass (Mackelprang et al., 2011; Frank-Fahle et al., 2014) and our results revealed that the storage temperature had no significant influence on VLP yields either from shallow or deep samples (Fig. 3), and this finding is consistent with results in aquatic systems and sediments (Brum, Schenck & Sullivan, 2013). Soil virus literature to date, suggests viral community structure is not different with depth (based on genetic material; Adriaenssens & Cowan, 2014), nor does abundance change (comparing 5–10 cm and 10–15 cm from soil pore water; Ballaud et al., 2015), but viral abundance was reported to vary seasonally (Ballaud et al., 2015). To this end, we tested storing both shallow and deep soil samples at chilled (4 °C) and frozen temperatures (−80 °C). In this experiment, the same protocol was followed from Experiment 1, but with AKC buffer and vortexing. Since there was not a significant difference in physical dispersion method from Experiment 1B, vortexing was chosen because bead-beating has been shown to release more humics from soil (i.e., ghost particles; Lakay, Botha & Prior, 2007) and the humic fragments might interfere with Amicon filter concentration (humics interact with minicolumns in Harry et al., 1999) and downstream processing of the samples (i.e., DNA extraction and sequencing; Saano et al., 1995; Zhou, Bruns & Tiedje, 1996).

Our results revealed that frozen samples had higher VLPs g^−1^ soil than chilled samples for shallow (Fig. 3A) and deep samples (Fig. 3B), but this difference was not significant (i.e., the lowest *p* value was 0.067 in the deep palsa sample). This general finding of no significant difference in VLP recoveries by storage temperate is consistent with results in aquatic systems and sediments (Brum, Schenck & Sullivan, 2013). One caveat is that these samples are Arctic samples, whose *in situ* habitat temperatures at the time of collection spanned “chilled” temperatures (∼0–15 °C at 36–40 cm depth, although ∼14–20 °C at 1–5 cm; the coldest conditions were in the deep palsa samples). Therefore, storage at 4 °C may allow microbial—and associated viral—communities (especially those at depth) to remain active, and thus drift from *in situ* composition, even though total viral recovery numbers did not change. In addition, while freeze-thaw cycles are known to broadly decrease infectivity for viruses (Ward et al., 2004), here the primary goal of recovery is enumeration and genomic sequencing, not culturing, and these viruses are also likely less sensitive to freeze-thaw given that it is a characteristic of their habitat. For these reasons, we conclude that frozen storage is the best choice for obtaining an unaltered viral community for these sample types.

The second part of this experiment tested an ecological difference rather than a methodological one: whether virus abundance changed with depth. Our frozen-storage data show significantly higher VLPs in deep than shallow palsa and fen (28% and 27% higher, respectively, *p* < 0.05; Fig. 3). Conversely, bog had higher virus abundance in the shallow samples (18%), although it was not significant. In the chilled samples, there was no significant difference between shallow and deep (Fig. 3); the trends were the same as for frozen samples for bog and fen, but opposite in palsa (shallow was 14% higher than deep).

In some aquatic systems virus abundance decreases with depth (Cochlan et al., 1993; Noble & Fuhrman, 1998; Parada et al., 2007), correlating with the decrease in microbial abundance with depth (Buitenhuis et al., 2012; Mediterranean Sea data in Mapelli et al., 2013). This is not surprising, as viral and bacterial abundances have been shown to be moderately correlated in at least some systems (Maranger & Bird, 1995; Anesio et al., 2004). If this trend also occurs in terrestrial environments, then we would expect viral abundance to decrease with depth, as it has been shown that microbial abundance decreases with depth (Mackelprang et al., 2011). In this study, this trend (viral abundance decreasing with depth) is supported only at the bog site (for both storage conditions) and at the palsa site with chilled storage. The palsa and bog habitats both have permafrost, with palsa’s being intact and the bog’s being partially thawed. The presence of permafrost would decrease microbial activity and thus viral activity (Frank-Fahle et al., 2014). However, it is contradicted at the palsa site with frozen storage and the fen site (for both storage conditions). At the palsa site, the depth increases in VLPs may be due to a concentration of virus particles and microbial cells due to the permafrost/active layer boundary for palsa samples, as has been observed for microbial cells at the permafrost boundary in other studies (permafrost in palsa starts ∼40 cm at Stordalen mire; reviewed in Jansson & Taş, 2014). At the fen site, which lacks permafrost, the depth increases in VLPs could be due to a temperature-stratification-based water density gradient in the inundated soil column (average fen temperature at 1–5 cm was 15.8 °C, and 13.5 °C at 30–36 cm). The difference in frozen versus chilled results for the palsa site may be due to the similarity of chilled storage to the *in situ* palsa deep conditions, due to its proximity to the permafrost table. Therefore, the stored chilled samples could be active and therefore likely to give artificial results. The frozen palsa results of higher VLP abundance at depth may represent *in situ* biological patterns whereas the chilled samples may be artificial and not represent the *in situ* viral communities. The chilled samples may instead reflect the viral communities in autumn or spring months when soil temperatures average ∼4 °C because that was the temperature at which these viruses were stored. The palsa habitat is not waterlogged (like a majority of bog habitats and all fen habitat at our site) and therefore the viruses may be more resistant to temperature change because soil has a lower heat capacity than water (Bristow, 1998).

### Experiment 3: optimization of concentration and purification methods

Having explored how buffer, dispersion, and sampling conditions impact viral yields (and used improvements therein to assess depth differences in viral abundances at the site), we next tested the efficacy of several concentration and purification methods. Specifically, we tested concentration on Amicon filters with and without BSA addition (for “blocking” of the membranes to decrease viral adhesion), followed by purification on a CsCl gradient.

**Figure 4:**
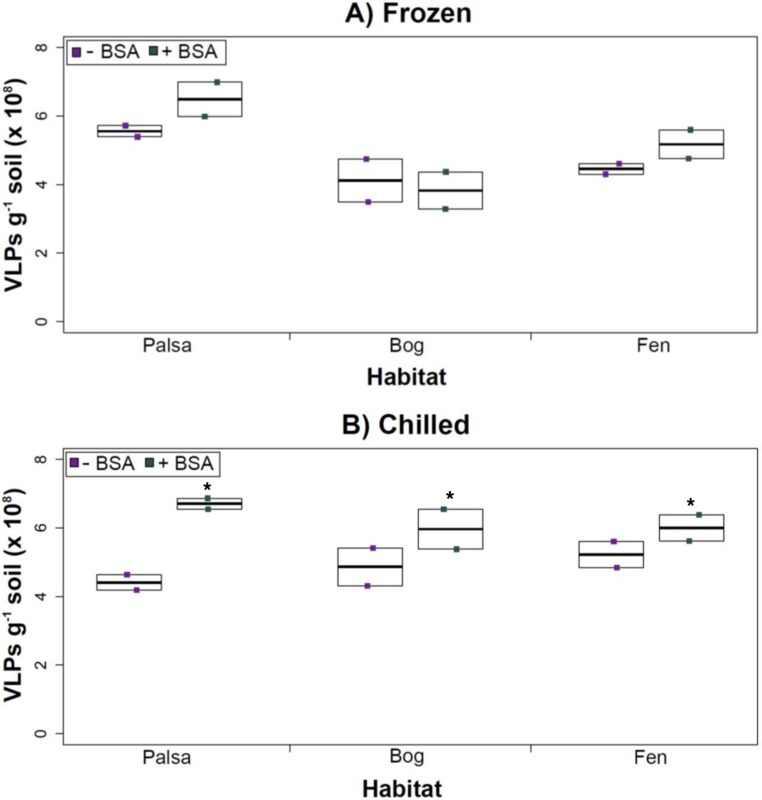
The impact of BSA on virus concentration by Amicon filter. Deep samples, stored frozen (A) or chilled (B), were used for this comparison and post concentration yields were counted by epifluorescence microscopy. An ^∗^ denotes statistically significant (*p* < 0.05) within the soil type.

The addition of BSA to Amicon filters quantitatively, though not significantly, increased viral yields (except bog stored frozen), but only significantly in chilled samples (Fig. 4). This is curious, as BSA has previously been shown to increase viral recovery yield by 2-fold with seawater viruses (Deng et al., 2012). We hypothesize that soil viruses across the three habitats in Stordalen mire may be less “sticky” than ocean viruses so that the blocking agent had little effect on yields off the Amicon filters (lack of “stickiness” of soil viruses characterized in Klitzke et al., 2015). This might reflect the highly charged soil environment, relative to seawater, selecting for reduced charges in soil virus tail fibers and structural proteins as compared to those in ocean viruses (Sobsey et al., 1980; Dowd et al., 1998). Additionally, soil viruses may be less “sticky” because they need to be able to attach to their host for infection and viral transport in soils is slow compared to marine environments because in marine environments viruses are transported by currents (Brum et al., 2015).

**Figure 5:**
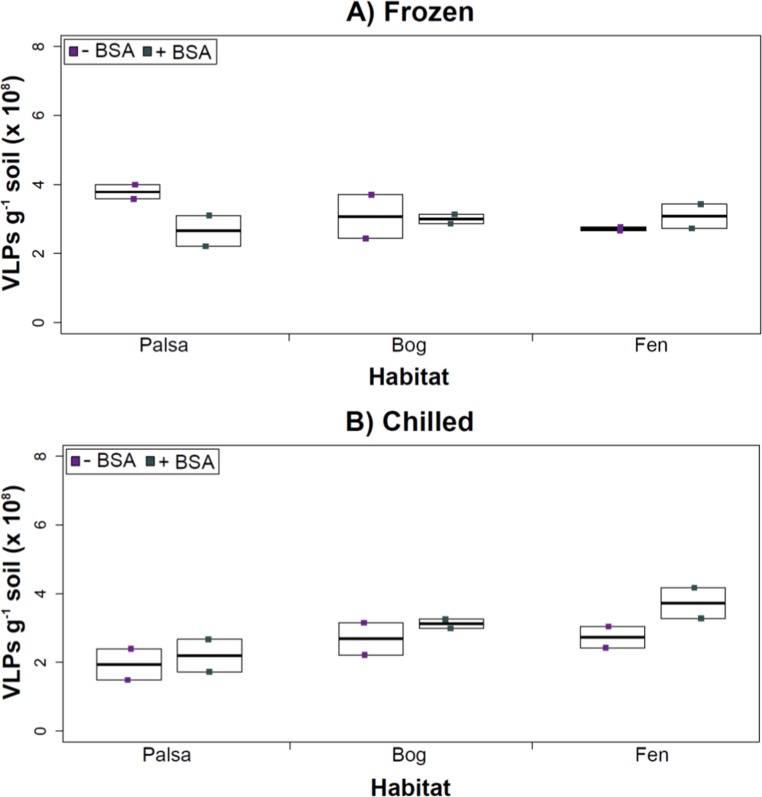
Comparison of samples with or without BSA after CsCl gradient purification. Counts were taken of deep samples that were CsCl gradient purified after Amicon filter concentration (with and without BSA) for samples from each habitat that were (A) frozen or (B) chilled.

Comparing samples with or without BSA in the Amicon step showed no significant effect after CsCl (although half had *p* values around 0.058; Fig. 5). Purification by CsCl gradient caused significant loss (*p* < 0.05, two-tailed paired *t*-test; Fig. S2) of VLPs in half of the samples, with 22–67% loss observed (Fig. S2).This loss is not surprising given that non-virus “ghost” particles such as humics and cell debris, which can be mistakenly counted as VLPs by EFM, are typically present in environments with high organic matter (e.g., as discussed in Suttle & Fuhrman, 2010; Forterre et al., 2013) and the CsCl densities (rho = 1.4–1.52 g/ml) used here are targeted to capture dsDNA viruses, while cell debris, humics, and ssDNA or RNA viruses migrate to lighter densities (Thurber et al., 2009). However, several of these assertions where addressed in other recent research (Williamson et al., 2013; Brum & Sullivan, 2015). The use of EFM, TEM, and flow cytometry enumeration techniques have been carefully examined and in each, the use of EFM was suggested (Hermes & Suttle, 1995; Weinbauer & Suttle, 1997; Noble & Fuhrman, 1998; Brum, Schenck & Sullivan, 2013; Williamson et al., 2013). The lowered VLP counts could be due to the CsCl damaging viral particles during purification, which is known to be osmotically challenging for many viral isolates. In seawater samples, where there is less concern for organic matter and ghost particle background, viral loss was observed from CsCl purification (Hurwitz et al., 2013). Also, others have observed viral recovery for isolates after CsCl purification to average between 53–66% with a range of 13–100% recovery (Chen, Estes & Ramig, 1992; Muniesa et al., 2004). Because seawater preparations are much cleaner and isolates are much less prone to VLP counts being augmented by organic matter and ghost particles, we would suggest that the reduced counts in CsCl purified soil virus communities are also likely legitimate VLP yield reductions. In summary, although CsCl gradients typically do represent “a loss step” they are important for purifying viral particles from contaminating nucleic acids or inhibitory substances for downstream processing (Saano et al., 1995; Zhou, Bruns & Tiedje, 1996), and outperform other purification options such as column chromatography or commercial purification kits (Duffy, O’Brien & Strappe, 2005).

## Conclusions

Accessing viruses in permafrost-associated peatland soils is a fundamental prerequisite for understanding their spatial and temporal dynamics, and more broadly peatland and permafrost-associated soil ecology. The three experiments here have identified an optimized protocol for viral resuspension from these high organic soils rich in humics: frozen storage conditions (chosen due to habitat considerations rather than a difference in recovery), AKC buffer with vortexing, concentration on Amicon filters with BSA, and purification via CsCl density gradient centrifugation. This optimized protocol can now be used to help further viral research in soil, particularly in challenging, humic-laden highly organic soils. At the same time, a revolution in viral ecology is being born out of experimental and informatic advances for studying viral signals at the single cell level (reviewed in Dang & Sullivan, 2014), in microbial datasets (Labonté et al., 2015; Roux et al., 2015a; Roux et al., 2015b) and through the development of quantitative sample-to-sequence pipelines for surveying viral communities (reviewed in Wommack et al., 2012; Solonenko & Sullivan, 2013; DeLong, 2013; Brum et al., 2016). The findings presented here, in parallel with these technical advances in viral ecology, should soon enable a more thorough understanding of viral diversity and viral impacts on permafrost-associated soil microorganisms, specifically impacts of viral infection upon host community structure, viral–host gene transfer, and viral influence on host cell physiology. These key findings can then feed into modeling efforts that should help elucidate the role(s) of viruses in biogeochemical cycles and, ultimately, improve our ability to incorporate them into climate change models.

## Supplemental Information

10.7717/peerj.1999/supp-1 Data S1 Raw data

10.7717/peerj.1999/supp-2 Figure S1 TEM micrographs of viruses and organic matter across a permafrost thaw gradient Micrographs of a virus from the bog (A and C) and fen habitats (B and D), and a micrograph of peat from the palsa habitat (E).

10.7717/peerj.1999/supp-3 Figure S2 CsCl Recovery of VLPs The recovery from CsCl purification was determined for deep samples across all three habitats. Samples were stored frozen without (A) or with (B) BSA or chilled without (C) and with BSA (D). An ∗ denotes statistically significant (*p* < 0.05) within the soil type.

## Additional Information and Declarations

### Data Availability

The following information was supplied regarding data availability: The raw data has been supplied as a Data S1.
