# Supplementary figures and images for: Optimization of viral resuspension methods for carbon-rich soils along a permafrost thaw gradient
Source: PeerJ. 2016 May 17;4:e1999. doi: 10.7717/peerj.1999 (PMC4878379; doi:10.7717/peerj.1999)

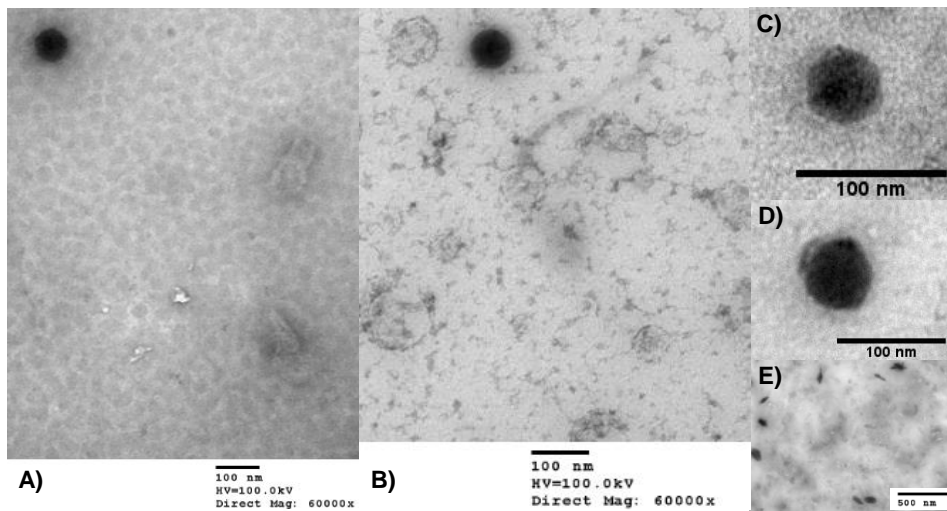

Supplement: Figure S1 — Micrographs of a virus from the bog (A and C) and fen habitats (B and D), and a micrograph of peat from the palsa habitat (E). [file peerj-04-1999-s002.pdf]

**A) Frozen samples**

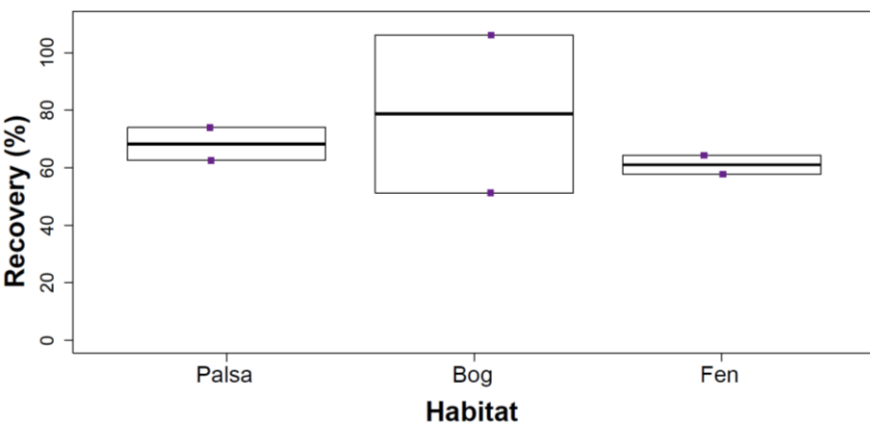

**B) Frozen samples + BSA**

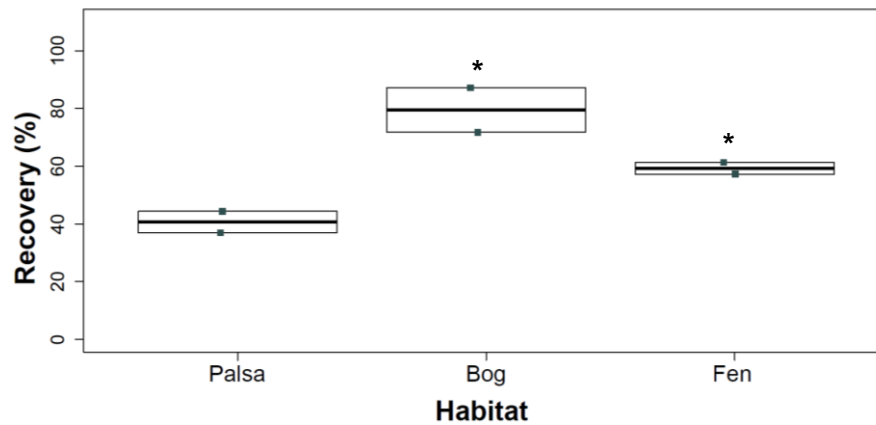

**C) Chilled samples**

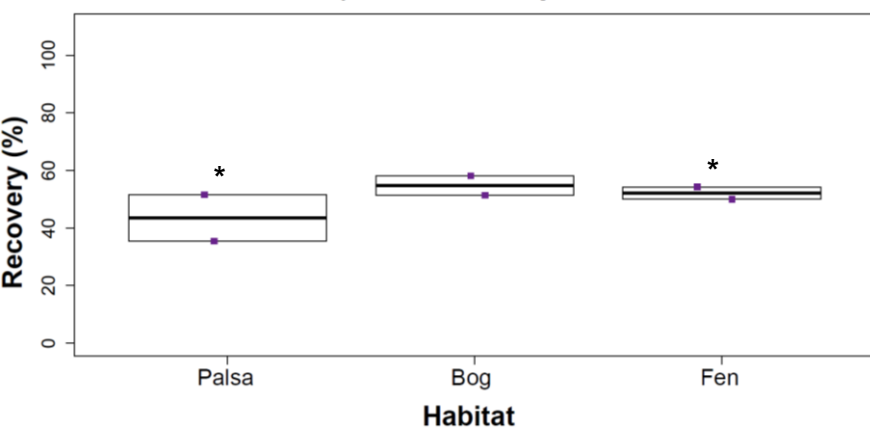

**D) Chilled samples + BSA**

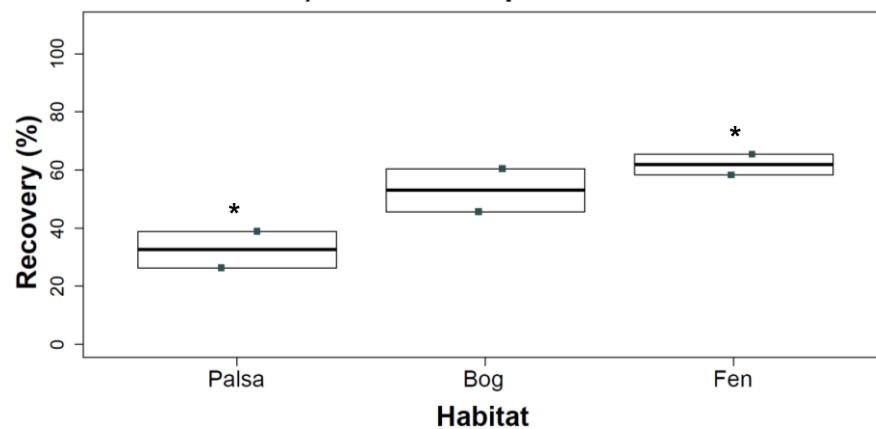

Supplement: Figure S2 — The recovery from CsCl purification was determined for deep samples across all three habitats. Samples were stored frozen without (A) or with (B) BSA or chilled without (C) and with BSA (D). An ∗ denotes statistically significant (p < 0.05) within the soil type. [file peerj-04-1999-s003.pdf]
